# Screening and evaluation of novel DPP-IV inhibitory peptides in goat milk based on molecular docking and molecular dynamics simulation

**DOI:** 10.1016/j.fochx.2025.102217

**Published:** 2025-01-30

**Authors:** Kuo Dang, Jing Lan, Yanli Wang, Daodong Pan, Lihui Du, Shikun Suo, Yali Dang, Xinchang Gao

**Affiliations:** a College of Food Science and Engineering, Ningbo University, Ningbo 315211, Zhejiang, China; b Institute of Drug Discovery Technology, Ningbo University, Ningbo 315211, China

**Keywords:** Goat milk, Virtual screening, *In vitro* simulated digestion, DPP-IV inhibitor, Inhibitory mode

## Abstract

Virtual screening techniques have gained much attention as a means of studying bioactive peptides. This study aimed to screen DPP-IV inhibitor peptides in goat milk after simulated digestion *in vitro* combined with molecular docking and dynamics simulations. By evaluating the docking energy and active sites, and by analyzing RMSD, RMSF, and Rg values, two novel peptides, GPFPLL and LPYPY, were successfully screened and identified. GPFPLL and LPYPY were found to exhibit high inhibitory activity against DPP-IV (IC_50_ of 130.68 ± 10.38 μM and 179.52 ± 18.89 μM, respectively). Both GPFPLL and LPYPY stably bound to S1 and S1’ in DPP-IV, and both demonstrated competitive inhibition of DPP-IV. The inhibition of DPP-IV by GPFPLL and LPYPY after *in vitro* digestion reached 31.90 % ± 1.80 % and 39.37 % ± 0.90 %, respectively. In a Caco-2 cell experiment, GPFPLL and LPYPY exhibited significant inhibition of DPP-IV, reaching 46.53 % ± 3.48 % and 65.98 % ± 2.87 %, respectively, when the concentration of each peptide was 2 mg/mL. The results of this study suggest that using molecular docking and dynamics simulations to screen novel peptides is an effective approach, and the identified peptides GPFPLL and LPYPY show potential for diabetes management.

## Highlights

• Molecular docking combined with MD for screening DPP-IV inhibitors.
• Novel DPP-IV inhibitory peptides GPFPLL and LPYPY were identified in goat milk.
• Both GPFPLL and LPYPY can exhibit stable binding to the S1 and S1’ of DPP-IV.
• GPFPLL and LPYPY demonstrate a competitive inhibition mode.
• GPFPLL and LPYPY retain high DPP-IV inhibitory activity during *in vitro* digestion.

## 1 Introduction

At present, there are more than 463 million people worldwide living with diabetes, and it is predicted that this number will reach 700 million by 2045, with the majority of diabetic patients suffering from type 2 diabetes mellitus (T2DM) (Chatterjee, Khunti, & Davies, 2018; Guo & Smith, 2021). The primary modalities for managing T2DM include dietary control, medication, and direct insulin injection. However, existing clinical data indicate that medication therapy is costly and may have potential side effects and may fail to achieve the desired outcomes in certain severe cases (Xu et al., 2019). Recently, bioactive peptides have been considered as a healthier and safer alternative. Dipeptidy1 peptidase-IV (DPP-IV) inhibitor peptides, which can effectively mitigate the diverse side effects of medications, such as nausea and abdominal discomfort caused by drugs, have received increasing attention for treating chronic diseases (Luo et al., 2022). Researchers have identified numerous bioactive peptides with various health benefits from foods, including milk, camel milk (Nongonierma et al., 2019), walnuts (Li, Guo, Chi, & Ma, 2020), coffee (Ribeiro, Rocha, & Prudencio, 2021), and chickpeas (Zan et al., 2023). Among them, milk-derived proteins have emerged as the most extensive source of bioactive peptides because of their diverse health benefits, including antioxidative (Lajnaf, Gharsallah, Jridi, Attia, & Ayadi, 2020), antidiabetic (Jia et al., 2020), antihypertensive (Villadóniga & Cantera, 2019), and immune-modulatory properties (Mohanty, Mohapatra, Misra, & Sahu, 2016).

Notably, goat milk has become an important source of protein and bioactive peptides due to its rich nutritional, physiological, and functional activities (Dhasmana, Das, & Shrivastava, 2022). Peptides from goat milk were shown to enhance insulin sensitivity and help treat type 2 diabetes (Gong et al., 2020). This effect was attributed to hypoglycemic peptides found in goat milk that had specific peptide sequences derived from κ-casein that exhibited high inhibitory activity against DPP-IV (Y. Zhang, Chen, Ma, & Chen, 2015). Therefore, efficiently mining the active ingredients in goat milk protein can both increase its economic value and societal benefits and promote the development of the dairy industry (Kostić et al., 2021) (Du et al., 2023). However, there is a relative paucity of research dedicated to screening and evaluating hypoglycemic peptides derived from goat milk and there has been limited research on the stability of DPP-IV inhibitory peptides in simulated *in vitro* digestion and changes in their DPP-IV inhibitory activity due to digestion.

Traditional methods for identifying bioactive peptides mainly rely on step-by-step separation techniques using multidimensional chromatography followed by peptide sequencing *via* LC/MS, which is a time-consuming and labor-intensive process (Ngoh & Gan, 2018). In contrast, computational methods provide a faster and more efficient approach for identifying bioactive peptides from a large pool of known peptides. For example, molecular docking technology has recently gained popularity because of its efficiency and cost-effectiveness for identifying bioactive peptides. Chen et al. (J. Chen, Yu, Chen, Wu, & He, 2022) used molecular docking to screen two novel angiotensin-converting enzyme (ACE) inhibitory peptides, EACF (IC_50_ of 41.06 μM) and CDF (IC_50_ of 192.17 μM). Similarly, Gao et al. (Gao et al., 2022) used molecular docking to screen a novel ACE inhibitory peptide, S-A (IC_50_ of 54.22 μM). However, molecular docking that relies on limited conformational sampling typically applies a “one-shot” approach to achieve binding conformations (W. Liu, Liu, Liu, Westerhoff, & Zheng, 2022). This method often disregards the overall flexibility of the receptor and ligand, reducing the accuracy of compound screening. A molecular dynamics simulation (MD) situates the receptor and ligand in a simulation environment similar to that of actual experimental conditions, enabling their free movement. This approach, which is based on continuous conformational sampling, overcomes the limitations of molecular docking (W. Liu et al., 2022). However, because of its significant computational costs, it is difficult to achieve rapid screening of bioactive peptides with MD. Therefore, the integration of molecular docking with MD capitalizes on the strengths of both methods while mitigating the limitations inherent in individual bioactive peptide screening techniques and enhances the interpretation of the resulting experimental data through the lens of molecular interactions.

In this study, we applied an integrated approach combining molecular docking with MD to identify peptides in a defatted goat milk digestive solution with potential DPP-IV inhibitory activity. The bioactivity of these peptides was validated through *in vitro* inhibition experiments and cell assays. In addition, the stability of the novel peptides in a digestive environment was investigated. Based on the combined screening strategy, this work contributes to uncovering novel DPP-IV inhibitory peptides in goat milk and provides theoretical support for exploring the potential of goat milk in functional foods and medical applications.

## 2 Materials and methods

### 2.1 Chemicals and reagents

Goat milk powder was purchased from a fresh supermarket located in Ningbo, Zhejiang Province, China. DPP-IV and Gly-Pro-pNA were obtained from Sigma-Aldrich Co., Ltd. (Shanghai, China). Sitagliptin, salivary amylase, gastric pepsin, and trypsin were obtained from Shanghai yuanye Bio-Technology Co., Ltd. (Shanghai, China). Caco-2 cells, basic culture medium (DMEM), non-inactivated fetal bovine serum, and penicillin-streptomycin were purchased from Haixing Biological Technology Co., Ltd. (Suzhou, China). A CCK-8 assay kit was obtained from Beijing Solarbio Science & Technology Co., Ltd. (Beijing, China). Gly-Pro-AMC was provided by the Shanghai Macklin Biochemical Technology Co., Ltd. (Shanghai, China). All other reagents were of analytical grade.

### 2.2 Preparation of defatted goat milk digestive solution

After the goat milk was mixed with ultrapure water at a ratio of 1:10 (m/v) at 45 °C, the diluted milk was centrifuged at 8000 rpm for 10 min, and the supernatant was collected to obtain defatted goat milk. The defatted goat milk was freeze-dried and stored at −80 °C for subsequent analysis (Du et al., 2022).

The freeze-dried defatted goat milk was prepared as a 50 mg/mL suspension with ultrapure water. The defatted goat milk was then mixed with salivary amylase (150 U/mL) and incubated at 37 °C for 5 min to simulate the oral phase of digestion. Subsequently, the pH was adjusted to 2.0 with HCI (6 M), and then pepsin (2000 U/mL) was added to simulate the gastric stage. The solution then incubated at 37 °C for 120 min. The pH was adjusted to 7.0 with NaOH (1 M), 100 U/mL trypsin was added, and the solution incubated at 37 °C for 120 min to simulate the intestinal stage. Once the reaction was complete, the enzyme was deactivated in a water bath at 100 °C for 10 min. Finally, the digested sample was collected through ultrafiltration tubes (3 kDa) and stored in a desiccator for subsequent analysis (Nongonierma et al., 2019).

### 2.3 Identification of peptides by LC-MS/MS

The digested sample (see Section 2.2) was analyzed by LC-MS/MS to identify the peptides. The system comprised a Thermoelectric Easy-nLC 1200 (Thermo Scientific) and an Orbitrap Exploris 480 (Thermo Scientific) for peptide identification. The parameters of the mass spectrometer were set in accordance with a previously described method (Zhang et al., 2022). The average local confidence (ALC%) was used as the confidence metric. Peptides with an ALC ≥ 90 % were considered reliable and were retained.

### 2.4 Potential bioactive peptide spectrum

The biological activity of the peptides was evaluated using the online tool PeptideRanker (http://distilldeep.ucd.ie/PeptideRanker/). PeptideRanker is a platform based on an advanced neural network model specifically designed to predict the potential bioactivity of the peptides (Coscueta, Batista, Gomes, da Silva, & Pintado, 2022). The model assigns a probability value to peptides within an activity prediction range of 0–1. Peptides with a score above 0.8 were identified as potential bioactive candidates for further screening. ToxinPred (https://crdd.osdd.net/raghava/toxinpred/) and AllerTOP (https://www.ddg-pharmfac.net/allertop/index.html) were also used to predict toxicity and evaluate the allergenicity of the peptides, respectively.

### 2.5 Virtual screening based on molecular docking

RDKit (2022.9.5) software (L. Wang, Zhang, Wang, Zhu, & Tan, 2021), implemented in Python (3.11.3), was used to generate peptide conformations based on their sequences. The built-in force fields MMFF94 and ETKDG in RDKit were used to generate a 3D conformation of the peptides and conduct energy minimization (Wang et al., 2023). The resulting MOL files containing the peptide molecular information were converted to PDB files using OpenBabel (3.1.1).

The human DPP-IV conformation (PDB id: 1WCY) (Hiramatsu et al., 2004) was obtained from the RCSB Protein Database (https://www.rcsb.org). Subsequently, PyMOL (2.6.0) was used to remove all water and co-crystallized compounds, perform hydrogenation, and set DPP-IV as a rigid receptor. Next, the ligands and receptors were prepared by merging nonpolar hydrogens, assigning Gasteiger charges, and eliminating lone pair electrons using SailVina (1.0). The torsions of all ligands were allowed to rotate. Molecular docking was performed using AutoDock Vina (1.2.5) using docking parameters slightly modified from a method described by Valenzuela Zamudio et al. (Valenzuela Zamudio, Hidalgo-Figueroa, Ortíz Andrade, Hernández Álvarez, & Segura Campos, 2022). The potential DPP-IV inhibitory peptides were screened by assessing their docking energy scores. The interactions between the peptides and protein were analyzed using Discovery Studio (2020). Finally, the results of the interaction forces and active sites were graphically presented using PyMOL and Discovery Studio.

### 2.6 Molecular dynamic simulation

Following the methodology described by Xiong et al. (Xiong et al., 2022) with minor adjustments, the optimal complex conformation obtained using molecular docking was subjected to MD using GROMACS (2019.06). Initially, the system was configured to the CHARMM36 force field, a cubic box was constructed, and the peptide-protein complex was placed at the center of the box and solvated by adding the TIP3P water model to the box. The system was neutralized by the addition of an appropriate quantity of ions. To guarantee the conformational integrity of the system, atomic overlap was avoided, and reasonable geometric configurations were maintained. The entire system underwent energy minimization treatment. NVT and NPT were used to equilibrate the system for 100 ps, with temperature coupling achieved through a V-scale thermostat and pressure coupling achieved through a Berendsen barostat. Subsequent MD simulation was then conducted for 80 ns, and the results were analyzed using an embedded program within GROMACS.

### 2.7 Synthesis of peptides

Following the screening results that identified peptides for subsequent analysis, the peptides were custom-synthesized by Apeptide Co., Ltd. (Shanghai, China). The purity of the peptides was determined to be at least 98 %, as confirmed by high-performance liquid chromatography (HPLC) analysis. Further details on the peptide synthesis can be found in the Supplementary materials (**Fig. S2, Fig. S3**).

### 2.8 DPP-IV inhibition by the peptides

The method outlined by Nongonierma et al. (Nongonierma & FitzGerald, 2013) was adapted for our study. Peptides were prepared at various concentrations and transferred to a 96-well microplate containing Gly-Pro-pNA (0.2 mM). The microplates were then incubated for 5 min. Subsequently, DPP-IV (0.0025 U/mL) was added, followed by an additional incubation for 60 min. The absorbance of the mixture was measured at 405 nm. The peptides, Gly-Pro-pNA, and DPP-IV were then dissolved in Tris-HCl (0.1 M). Tris-HCl was used as the negative control with DPP-IV but without the peptides and as the blank control without the peptides or DPP-IV. Sitagliptin was used as the positive control instead of the peptides. The formula for calculating the inhibitory activity was as follows:

$$\mathrm{DPP}-\mathrm{IV}\;\text{inhibitory activity}\;\left(\%\right)=\left[\left(A_{c}-A_{s}\right)/\left(A_{c}-A_{b}\right)\right]\times 100$$

where absorbance values for the control, sample, and blank groups were designated as A_c_, A_s_, and A_b_, respectively.

### 2.9 *In vitro* simulated digestion of peptides

Based on the method described by Brodkorb et al. (Brodkorb et al., 2019), the following solutions were prepared: SSF, SGF, and SIF salt solutions, and salivary amylase, pepsin, and trypsin solutions.

#### 2.9.1 Oral phase

Each peptide was dissolved at a concentration of 1 mg/mL, and 0.094 mL of SSF, 0.001 mL of CaCl_2_·2H_2_O (0.3 M), 0.025 mL of salivary amylase (75 U/mL), and 0.005 mL of H_2_O were sequentially added, for a total volume of 0.25 mL. The mixture was then incubated in a 37 °C incubator for 2 min.

#### 2.9.2 Gastric phase

Briefly, 0.25 mL of the oral digestion mixture was sequentially combined with 0.12 mL of SGF, 0.001 mL of CaCl_2_·2H_2_O (0.3 M), 0.048 mL of pepsin (2000 U/mL), 0.060 mL of SGF, 0.011 mL of H_2_O, and 0.010 mL of HCl (1 M), for a total volume of 0.5 mL. The mixture was then incubated in a 37 °C incubator for 2 h.

#### 2.9.3 Intestinal phase

A 0.50-mL sample of the gastric digestion mixture was successively combined with 0.195 mL of SIF, 0.001 mL of CaCl_2_·2H_2_O (0.3 M), 0.125 mL of trypsin (100 U/mL), 0.100 mL of SIF, and 0.079 mL of H_2_O. The mixture was then incubated in a 37 °C incubator for 2 h at a pH of 7.0.

After oral, gastric, and intestinal digestion, the digestion mixture was filtered through a 0.22-μm sterile filter. The filtrate was stored at −20 °C for subsequent analysis.

#### 2.9.4 Stability of peptides after digestion

The *in vitro* simulated digestion experiments of the peptides were conducted in accordance with the method described by Hao et al., with minor modifications (Hao et al., 2020). The stability of peptides post-digestion was assessed by RP-HPLC (CAP-CELL PAK C18 AQ S-5, 10 mm × 150 mm column, Shiseido, Tokyo, Japan) with a detection wavelength of 228 nm. The formula for determining the peptide stability was as follows:

$$\begin{array}{c} \text{Stability of peptides}\;\left(\%\right)=\left(P_{a}/P_{b}\right)\times 100 \end{array}$$

where P_a_ represents the peak area of the digested sample, and P_b_ represents the peak area of the reference sample.

### 2.10 Cytotoxicity assay

A peptide cytotoxicity assay using Caco-2 cells was conducted based on the method outlined by Hao et al. with minor modifications (Hao et al., 2020). Initially, the cells were evenly seeded on a 96-well plate with 100 μL of 2 × 10^4^ cells. After seeding, the plate was placed in a sterile and CO_2_-enriched incubator. After a 24-h incubation, the medium was removed, and the adherent cells were used for subsequent experiments. The peptides were dissolved in the basal medium (DMEM) and cells were treated with different concentrations of peptides. After a 24-h incubation period, 10 μL of CCK-8 was added to each well and incubated with the cells. The absorbance at 450 nm was recorded to determine cell viability.

The following formula was used to calculate cell viability, with cells cultured in the basal medium used as the control group for comparison:

$$\begin{array}{c} \text{Cell viability}\left(\%\right)=\left(A_{\text{sample}}/A_{\text{control}}\right)\times 100 \end{array}$$

### 2.11 DPP-IV inhibitory activity in Caco-2 cells

Caco-2 cells were seeded on a black 96-well plate at a density of 2 × 10^4^ cells per well. Subsequently, the cells were treated with sample solutions at various concentrations and then further incubated for 24 h. After incubating, the peptide solutions and medium were removed from the wells, and 100 μL of the Gly-Pro-AMC substrate (50 μM) was added to each well and incubated for 10 min at 37 °C. Sitagliptin was used as the positive control instead of the peptide. The fluorescence intensity was measured at 350 nm and 440 nm using a multifunctional microplate reader (You et al., 2022).

### 2.12 Statistical analysis

All experiments were conducted independently. Statistical analyses were performed using SPSS25 (Chicago, USA). Before the difference comparison, normality and variance homogeneity tests were carried out. This confirmed that the data distribution adhered to normality, and the variance among the data groups was homogeneous. One-way analysis of variance (ANOVA) and post-hoc comparisons *via* the Tukey test were used to compare differences among groups with a significance of *p* ≤ 0.05. All graphical presentations were prepared using GraphPad Prism 9.5.1 (CA, USA).

## 3 Results and discussion

### 3.1 DPP-IV inhibitory peptide screening

A previous study showed that after hydrolyzing defatted goat milk with papain, the components with a molecular weight less than 3000 Da exhibited superior DPP-IV inhibition compared to the other components (85.54 % inhibition at 35.12 mg/mL) (Du et al., 2022). Interestingly, we determined that the defatted goat milk that underwent simulated digestion *in vitro* enhanced DPP-IV inhibition (IC_50_ = 10.22 ± 0.72 mg/mL) (**Fig. S1**). Consequently, peptidomics and virtual screening techniques were then used to search for DPP-IV inhibitory peptides in the digestion samples.

In the bioactive peptide library BIOPEP-UWM (https://biochemia.uwm.edu.pl/en/biopep-uwm-2/), nearly 80 % of the DPP-IV inhibitory peptides are of 3–7 amino acids in length. Therefore, peptides of this length with a confidence level of at least 90 % were screened from samples of the digested defatted goat milk. To enhance the accuracy of screening, the biological activity of each peptide was evaluated using the bioactivity prediction platform PeptideRanker. Peptides with scores greater than 0.8 were selected, and ultimately 38 potentially bioactive peptides were identified. Molecular docking was performed on the 38 peptides, which were ranked in descending order based on their binding energy. The first four peptides, GPFPLL (GL-6), LPYPY (LY-5), GPFPILN (GN-7), and RPWR (RR-4), were chosen for subsequent analysis (Table 1).

**Table 1:** Probability of activity prediction, binding energy, and physicochemical properties of 38 peptides.

Sequence	PeptideRanker	Binding Energy (kcal/mol)	Toxicit	Allergy
LPYPY	0.829798	−9.3	Non-toxic	Non-allergic
GPFPILN	0.905623	−9.2	Non-toxic	Non-allergic
RPWR	0.938535	−9.0	Non-toxic	Non-allergic
GPFPLL	0.967691	−8.8	Non-toxic	Non-allergic
FYPQLFRQ	0.843689	−8.7	Non-toxic	Non-allergic
WHLR	0.829836	−8.7	Non-toxic	Non-allergic
FALPMH	0.827399	−8.7	Non-toxic	Non-allergic
WFPK	0.978116	−8.7	Non-toxic	Non-allergic
GPFPL	0.974291	−8.7	Non-toxic	Non-allergic
GPFPILV	0.869285	−8.7	Non-toxic	Non-allergic
FIFPPKPK	0.822883	−8.7	Non-toxic	Non-allergic
TFAFR	0.812138	−8.7	Non-toxic	Non-allergic
LFRYL	0.869535	−8.6	Non-toxic	Non-allergic
LPRPLL	0.845233	−8.6	Non-toxic	Non-allergic
WKPW	0.980005	−8.5	Non-toxic	Non-allergic
RLFR	0.847988	−8.5	Non-toxic	Non-allergic
PLFR	0.92776	−8.4	Non-toxic	Non-allergic
APFPEVFR	0.858066	−8.4	Non-toxic	Non-allergic
FEMPFPK	0.901993	−8.3	Non-toxic	Non-allergic
LLRPFL	0.891102	−8.3	Non-toxic	Non-allergic
VSQPPFMP	0.866536	−8.3	Non-toxic	Non-allergic
PQLFR	0.855427	−8.3	Non-toxic	Non-allergic
AMKPWL	0.920568	−8.2	Non-toxic	Non-allergic
GPFPLLV	0.884211	−8.2	Non-toxic	Non-allergic
FPPKPK	0.816796	−8.1	Non-toxic	Non-allergic
ALPMHIRL	0.811703	−8.1	Non-toxic	Non-allergic
QPSPFMP	0.953185	−8	Non-toxic	Non-allergic
FMALPPK	0.813184	−8	Non-toxic	Non-allergic
AMKPWR	0.888884	−7.9	Non-toxic	Non-allergic
LMFPK	0.858524	−7.9	Non-toxic	Non-allergic
MFFR	0.990491	−7.8	Non-toxic	Non-allergic
FAMKPW	0.939504	−7.8	Non-toxic	Non-allergic
VPPF	0.926314	−7.8	Non-toxic	Non-allergic
MPFPK	0.933075	−7.7	Non-toxic	Non-allergic
PPPKP	0.857011	−7.7	Non-toxic	Non-allergic
FLPK	0.846584	−7.6	Non-toxic	Non-allergic
LPLPL	0.816409	−7.5	Non-toxic	Non-allergic
MKPW	0.931763	−7.4	Non-toxic	Non-allergic

Molecular docking was used to investigate binding between the peptides and DPP-IV. A lower energy value implies a more stable molecular binding conformation (Lagunes et al., 2019). IPI was used as a positive control for molecular docking as it is considered the most powerful DPP-IV inhibitory peptide (Nongonierma & FitzGerald, 2019). Interestingly, GL-6 (−8.8 kcal/mol), LY-5 (−9.3 kcal/mol), GN-7 (−9.2 kcal/mol), and RR-4 (−9.0 kcal/mol) all exhibited a lower docking energy with DPP-IV than did IPI (−7.2 kcal/mol). This indicated that all four peptides had the potential to inhibit DPP-IV. Xu et al. showed that the docking energy range of the Gly-Pro DPP-IV inhibitory peptide derived from collagen was between −5.6 and − 7.5 kcal/mol. (Xu, Zheng, Huang, & Zhao, 2024a). Zan et al. reported that the docking energy of peptides extracted from chickpeas with DPP-IV ranged from −5.6 to −8.3 kcal/mol (Zan et al., 2023). Nongonierma et al. found that the docking energy scores of milk protein-derived peptides and DPP-IV ranged from −5.5 to −10.8 kcal/mol (Nongonierma, Mooney, Shields, & FitzGerald, 2014). These energy values were within a range in which stable interactions occur and were consistent with our computational outcomes. This indicated that all four peptides had the potential to interact with DPP-IV and exhibit DPP-IV inhibitory activity (Ji et al., 2020). Studies have demonstrated that the Vina score of a peptide correlate to a degree with its DPP-IV inhibition, but this cannot be the sole basis for determining inhibitory activity. This is because molecular docking is unable to distinguish between the substrate and the inhibitor (M. Zhang et al., 2022). Therefore, further analysis was necessary to evaluate peptide binding in the active site of DPP-IV.

**Fig. 1:**
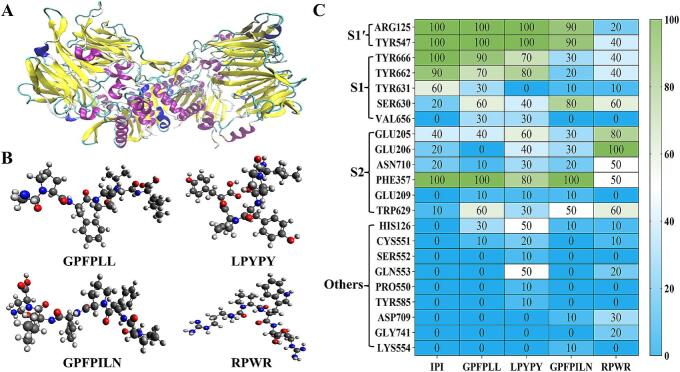
The 3D conformations of DPP-IV and four screened peptides (A, B). A: DPP-IV; B: Peptides. Heat map of the frequency of docking sites in ten docking poses (C). (S1’, S1, and S2 represent different active binding regions of DPP-IV and the amino acid residues in Others were not located in the active binding regions).

The molecular architecture of DPP-IV comprises two structural domains, the C-terminal α/β hydrolase active site region and the N-terminal eight-bladed β-propeller domain, which accommodates four active binding regions: S1 (Tyr662, Tyr666, Val656, Ser630, and Tyr631), S1’ (Arg125, Tyr547, and His740), S2 (Glu205, Glu206, and Asn710), and S2 extensive (Val207, Ser209, Phe357, Trp629, and Arg358) (Juillerat-Jeanneret, 2014; L. Wang, Ding, Du, Yu, & Liu, 2019). The 3D conformations of DPP-IV and the four screened peptides are depicted in Fig. 1A, B. Ten docking poses of IPI-DPP-IV, GL-6-DPP-IV, LY-5-DPP-IV, GN-7-DPP-IV, and RR-4-DPP-IV were used to analyze the frequency of active sites (Fig. 1C). When bound to IPI, the residues ARG125 and TYR547 in S1’, TYR666 and TYR662 in S1, and PHE357 in S2 remained stable in 10 docking poses. These residues were shown to be necessary for the coordination of substrates, and interaction between these residues and inhibitors can hinder the catalytic degradation of substrates by DPP-IV (Aertgeerts et al., 2004). Hydrogen bond forces and hydrophobic forces are generally considered to be the main interactions between DPP-IV inhibitor peptides and DPP-IV, with hydrogen bonds providing an indispensable role (You et al., 2022). Xu et al. discovered that residues ARG125 and TYR547 in DPP-IV formed stable hydrogen bonds with peptides that had Pro at the second N-terminal position, and residues TYR666, TYR662, and PHE357 generated stable hydrophobic forces with the peptides (Xu, Zheng, Huang, & Zhao, 2024b). These features accounted for the strong DPP-IV inhibitory activity of IPI. Interestingly, both GL-6 and LY-5 could stably bind to ARG125, TYR547, TYR666, TYR662, and PHE357 at 10 docking poses, which was similar to that of IPI. However, GN-7 did not stably bind to TYR662 or TYR666, and RR-4 did not stably bind to any of the key residues at 10 docking poses. The molecular docking results showed that GL-6 and LY-5 stably bound to the active site of DPP-IV, thereby demonstrating their potential as strong inhibitors of DPP-IV (Mojica & de Mejía, 2016).

### 3.2 Analysis of molecular dynamics simulation results

**Fig. 2:**
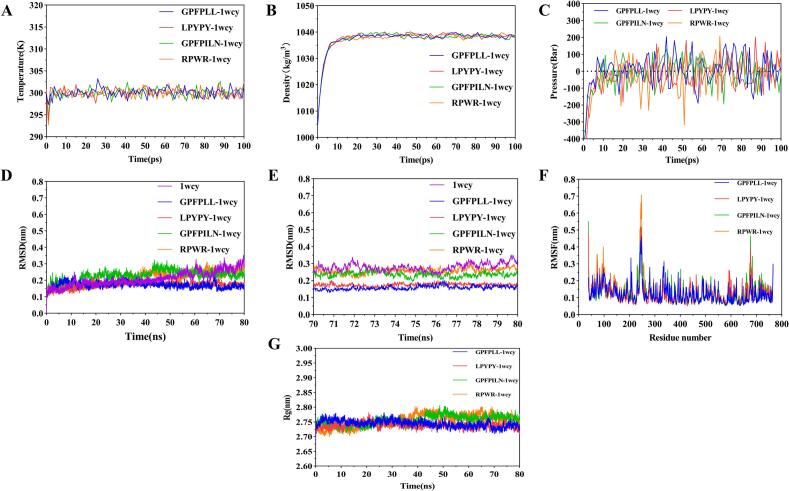
Changes in parameters during the NVT and NPT equilibrium processes of peptide-protein complexes within 100 ps (A-C) and stability indicators for molecular dynamics simulation (D-G). A: Temperature; B: Density; C: Pressure. D: RMSD values of the peptide-protein complexes formed within the 80 ns molecular dynamics simulation system; E: RMSD values of the peptide-protein complexes formed during the 70–80 ns timeframe; F: Rg values of DPP-IV within the 80 ns molecular dynamics simulation system; G: RMSF values of DPP-IV within the 80 ns molecular dynamics simulation system.

MD can evaluate the stability and interactions of an enzyme-ligand complex from a dynamic viewpoint (Jin & Wei, 2024). To further assess the potential of GL-6, LY-5, GN-7, and RR-4 to form stable complexes with DPP-IV, MD simulations were performed to visualize the atomic-level binding dynamics. Before the simulation, the system was equilibrated by applying the NVT and NPT ensembles to ensure that the internal temperature, pressure, and density of the system attained thermodynamic equilibrium (Fig. 2A–C). The stability of each peptide-protein complex was evaluated using the root-mean-square deviation (RMSD), root-mean-square fluctuation (RMSF), and radius of gyration (Rg).

#### 3.2.1 Root-mean-square-deviation analysis

The dynamic stability of GL-6, LY-5, GN-7, and RR-4 with the receptor protein DPP-IV was evaluated using RMSD. The RMSD value indicates the conformational stability of the peptide-protein complex (Mehmood et al., 2022). Complexes GL-6-1WCY and LY-5-1WCY reached equilibrium after 20 ns, and GN-7-1WCY and RR-4-1WCY reached equilibrium after 45 ns and 60 ns, respectively (Fig. 2D). By analyzing the trend in the fluctuations during the last 10 ns of the simulation (70–80 ns), the average RMSD values of GL-6-1WCY, LY-5-1WCY, GN-7-1WCY, and RR-4-1WCY were calculated to be 0.1580 ± 0.0110 nm, 0.1753 ± 0.0083 nm, 0.2359 ± 0.0146 nm, and 0.2580 ± 0.0158 nm, respectively (Fig. 2E). The RMSD values of the complexes formed by these four peptides with DPP-IV were all lower than the previously reported RMSD value of the DPP-IV inhibitory peptide VPLVM with DPP-IV (Pei et al., 2022), indicating that all four peptides stably bound to DPP-IV. GL-6 and LY-5 bound more stably with DPP-IV than did GN-7 and RR-4, implying that GL-6 and LY-5 were DPP-IV inhibitor candidates. Similar results were reported by Qi et al. (Qi et al., 2022), who found that the average RMSD value of a complex between the peptide LLW and hypoxanthine oxidase was lower than that of a complex between the peptide PEW and hypoxanthine oxidase, indicating that LLW formed a tighter complex with hypoxanthine oxidase.

#### 3.2.2 Radius-of-gyration analysis

The Rg is commonly used as an indicator to describe the compactness of a protein conformation, with lower values reflecting higher stability (J. G. Chen, Wu, Zhang, Yin, & Zhang, 2020; Q. Wang, Wang, & Chen, 2016). As shown in Fig. 2G, the average Rg values of GL-6-1WCY, LY-5-1WCY, GN-7-1WCY, and RR-4-1WCY were 2.744 ± 0.011 nm, 2.739 ± 0.009 nm, 2.755 ± 0.016 nm, and 2.755 ± 0.022 nm, respectively. This suggested that under the influence of GP-6 or LY-5, the conformation of DPP-IV became more compact. Wang et al. also discovered that the conformation of α-glucosidase was more compact under the influence of FAPSW in a 50-ns simulation system (Wang et al., 2023).

#### 3.2.3 Root-mean-square fluctuation analysis

The RMSF value is a measure of the degree of fluctuation in amino acid residues within a protein, in which higher values imply greater fluctuations (Zhou, Bie, Li, Yuan, & Zhou, 2023). Therefore, the RMSF is often used to assess the flexibility of residues, which can reveal local changes in the protein conformation (Y. Liu et al., 2021). The average RMSF values of the complexes GL-6-1WCY, LY-5-1WCY, GN-7-1WCY, and RR-4-1WCY were 0.122 ± 0.054 nm, 0.124 ± 0.059 nm, 0.129 ± 0.062 nm, and 0.136 ± 0.076 nm, respectively (Fig. 2F). These findings suggested that these four peptides could form stable complexes with 1WCY and that the conformations of GL-6-1WCY and LY-5-1WCY were more stable than those of GN-7-1WCY and RR-4-1WCY. Du et al. identified four DPP-IV inhibitory peptides in goat whey protein, and the peptides with stronger DPP-IV inhibitory activity showed smaller fluctuations in their RMSF values. This was consistent with our results (Du et al., 2023).

Thus, GL-6, LY-5, GN-7, and RR-4 formed stable complexes with DPP-IV and exhibited potential inhibitory capabilities. Furthermore, the inhibitory activity of GL-6 and LY-5 against DPP-IV was potentially superior to that of GN-7 and RR-4.

### 3.3 Validation of DPP-IV inhibition by the peptides

**Fig. 3:**
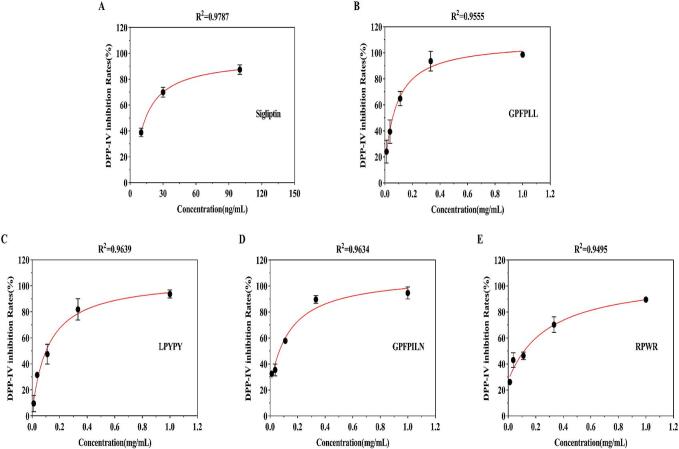
DPP-IV inhibition rates at different concentrations, along with the nonlinear fitting curves (A-E). A: Sitagliptin; B: GPFPLL; C: LPYPY; D: GPFPILN; E: RPWR.

Molecular docking and MD simulations have confirmed that GL-6 and LY-5 formed stable complexes with the receptor and potentially inhibited DPP-IV activity. Next, we confirmed the inhibitory activity of GL-6 and LY-5 against DPP-IV through further experimental verification. Therefore, the four peptides were chemically synthesized, and their inhibitory activity was evaluated through *in vitro* experiments. Sitagliptin is an effective DPP-IV inhibitor, and it has been widely used as a positive control for assessing DPP-IV activity *in vitro* (Scott, 2017). The IC_50_ of sitagliptin for DPP-IV inhibition was 0.02 ± 0.01 μM, which is consistent with the results of Vawhal et al. (Vawhal et al., 2023). The IC_50_ values of GL-6 and LY-5 were 130.68 ± 16.38 μM and 179.52 ± 18.89 μM, respectively (Table 2 and Fig. 3). Chen et al., (H. H. Chen et al., 2023) identified DPP-IV inhibitory peptides from hemp seed protein with IC_50_ values ranging from 80 μM to 290 μM (LPQNIPPL, YPYY, YPW, WWW, and YPY). Du et al., (Du et al., 2023) reported DPP-IV inhibitory peptides from goat milk papain hydrolysates with IC_50_ values ranging from 56.22 μM to 200.35 μM (SPPEFLR, FNPTY, YPVEPFT, and GPPEHLR). Nongonierma et al., (Nongonierma et al., 2019) identified DPP-IV inhibitory peptides from camel milk with IC_50_ values ranging from 55.10 μM to 330.10 μM (VPF, LAHKPL, and EPVK). You et al., (You et al., 2022) reported DPP-IV inhibitory peptides from quinoa protein with IC_50_ values ranging from 148.27 μM to 210.90 μM (IPIIN, APW, IPAV, and IPF). Mu et al., (Mu et al., 2024) identified DPP-IV inhibitory peptides from walnut protein with IC_50_ values ranging from 215.60 μM to 959.40 μM (LPFA, VPFWA, WGLP, FWVP, AFEP, FAVP, PLPW, GGF, RW, and GFR). Therefore, these results demonstrated that GL-6 and LY-5 exhibited significant inhibitory effects on DPP-IV *in vitro*.

**Table 2:** DPP-IV inhibition capacity of 4 peptides and sigliptin.

Number	Sequence	DPP-IV inhibitory activity IC_50_ (μM)
1	GPFPLL	130.68 ± 10.38
2	LPYPY	179.52 ± 18.89
3	GPFPILN	198.18 ± 14.90
4	RPWR	474.17 ± 14.34
5	Sigliptin	0.02 ± 0.01

The presence of Pro at the second position from the N-terminus of peptides has been shown to be crucial for blood sugar control (Taga, Hayashida, Kusubata, Ogawa-Goto, & Hattori, 2017). This was also observed for DPP-IV inhibitory peptides from other sources, including camel milk protein hydrolysate (LPVP) (Nongonierma, Paolella, Mudgil, Maqsood, & FitzGerald, 2018), bovine milk protein hydrolysate (LPVPQ) (Nongonierma & FitzGerald, 2016), bovine collagen protein hydrolysate (VPVG) (He et al., 2023), and chickpea protein hydrolysate (PPGIPYW) (Zan et al., 2023). This requirement is consistent with GL-6, LY-5, GN-7, and RR-4, all exhibiting inhibitory activity against DPP-IV. Interestingly, although both GN-7 and RR-4 exhibited the patterns described above, their DPP-IV inhibitory activity was not as strong as that of GL-6 and LY-5. First, GL-6 and LY-5 stably interacted with the key residues of DPP-IV, specifically ARG125, TYR547, TYR662, TYR666, and PHE357, across 10 docking poses. Second, relatively low RMSD, RMSF, and Rg values, along with low fluctuations, typically suggest the high conformation stability of the protein complex (Tripathi, Ganeshpurkar, Singh, & Krishnamurthy, 2023). The difference in the DPP-IV inhibitory activity of the four peptides may be attributed to the lower RMSD, RMSF, and Rg values of DPP-IV-GL-6 and DPP-IV-LY-5 compared to DPP-IV-GN-7 and DPP-IV-RR-4. These results all indicated that the complexes formed by GL-6 and LY-5 with DPP-IV were more stable than those formed by GN-7 and RR-4 with DPP-IV.

### 3.4 Inhibitory mode of peptides

**Fig. 4:**
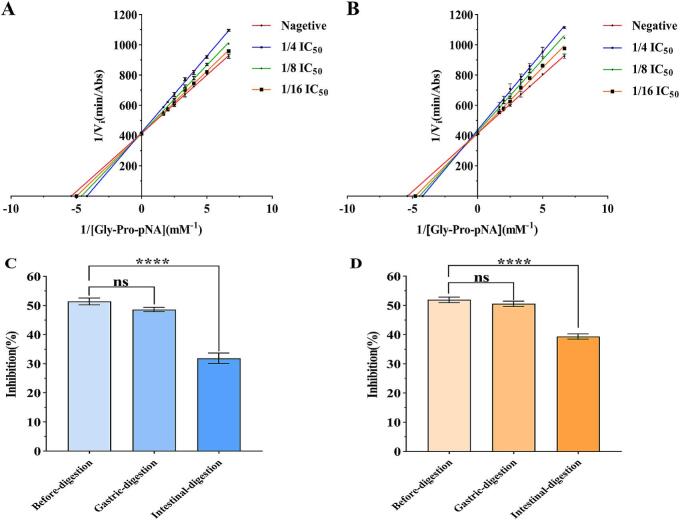
Lineweaver−Burk double-reciprocal plots for DPP-IV inhibitory peptides (A-B) and the inhibitory DPP-IV activity changes during the process of digestion (C—D). A, C: GPFPLL B, D: LPYPY.

Some studies have demonstrated that the high inhibitory activity of peptides may stem from their competitive inhibition of DPP-IV (Yu, Wang, Shuian, Liu, & Zhao, 2023). A Lineweaver–Burk double reciprocal plot (Xu et al., 2024a) was used to determine the mode of inhibition of GL-6 and LY-5. Competitive inhibition of GL-6 and LY-5 was clearly observed (Fig. 4A, B). These results were consistent with the conclusion that peptides with Pro as the second N-terminal residue may competitively inhibit DPP-IV (Hong et al., 2020). The peptide PPGIPYW, isolated from chickpea protein, exhibited remarkable DPP-IV inhibitory activity through competitive inhibition (Zan et al., 2023). Similarly, the peptide IPYWTY, isolated from pea protein, showed strong DPP-IV inhibitory activity and was also confirmed to act through competitive inhibition (Zhang, Zhu, et al., 2022). The peptide LPFYPN, isolated from coix seed protein, exhibited high DPP-IV inhibitory activity, also through competitive inhibition (S. Zhang et al., 2023). Previous studies demonstrated that peptides with Pro at the second N-terminal position were cleaved by DPP-IV, and the resulting hydrolysates continued to inhibit DPP-IV, which might explain the competitive inhibitory inhibition of GL-6 and LY-5 (Xu et al., 2019).

### 3.5 *In vitro* simulated digestion of peptides

Because of hydrolysis by digestive enzymes in the body, such as pepsin and trypsin, peptides are easily degraded during digestion, thereby reducing their bioactivity (Duffuler, Bhullar, de Campos Zani, & Wu, 2022). Therefore, it is essential to determine the stability of peptides by *in vitro* simulated digestion. As shown in Table 3, the stability of GL-6 and LY-5 after oral digestion was 99.60 % and 99.49 %, respectively. After gastric digestion, their stability remained high at 96.37 % and 97.60 %, respectively. This indicated that GL-6 and LY-5 retained high biological activity after oral and gastric digestion. Nevertheless, after intestinal digestion, the stability of GL-6 and LY-5 decreased by 26.89 % and 15.61 %, respectively. This may have been because the degradation of these peptides typically occurs during the intestinal phase of digestion (Fan & Wu, 2021). Trypsin in the intestine led to significant peptide degradation, which preferentially cleaved the amino acid residues tryptophan, leucine, aspartic acid, tyrosine, and isoleucine, thereby reducing the biological activity of the peptides (Vreeke, Vincken, & Wierenga, 2023). These findings were analogous to those of Yin et al. (Yin et al., 2022), who discovered that the stability of LLSPP and LPVGP decreased to 80 % after undergoing intestinal digestion. The reason that LY-5 exhibited better gastrointestinal resistance than GL-6 can also be explained. First, GL-6 is longer than LY-5, and longer peptides typically have poorer gastrointestinal resistance. Second, hydrophobicity predictions of GL-6 and LY-5 were conducted using the online software Expasy (http://web.expasy.org/protparam/), and the grand average of hydropathicity (GRAVY) of GL-6 and LY-5 was 1.133 and − 0.40, respectively. Other studies demonstrated that highly hydrophobic peptides exhibit poor stability during gastrointestinal digestion (B. Wang, Xie, & Li, 2019). LY-5 is notably less hydrophobic than GL-6, which may be the reason that LY-5 demonstrated higher stability during gastrointestinal digestion. These results suggested that the peptides GL-6 and LY-5 exhibited high resistance to oral and gastric digestion, but they exhibited relatively weak resistance to intestinal digestion.

**Table 3:** Digestive stability of peptides GPFPLL and LPYPY at different stages of the oral, gastric, and intestinal digestion.

Sequence	Mass (Da)	Oral digestion (%)	Oral-gastric digestion (%)	Oral-gastric-intestinal digestion (%)
GPFPLL	642.78	99.60	96.37	69.48
LPYPY	651.75	99.49	97.60	81.99

In addition, as shown in Fig. 4C, D, the DPP-IV inhibitory activity of GL-6 (at a concentration of 130.68 μM) and LY-5 (at a concentration of 179.52 μM) did not significantly decrease after oral-gastric digestion (*p* > 0.05). This was consistent with the previous results, in which there was no significant change in the stability of GL-6 and LY-5 after oral-gastric digestion. Interestingly, although the DPP-IV inhibitory activity of GL-6 and LY-5 decreased significantly after intestinal digestion, the peptides still retained a considerable inhibitory effect, with inhibitory rates of 31.90 ± 1.80 % and 39.37 ± 0.90 %, respectively. Wang et al. also found that α-glycosidase inhibition of FAPSW and MPGPP did not decrease significantly after oral-gastric digestion compared with that before digestion. However, the inhibition of α-glycosidase by the peptides decreased significantly after intestinal digestion, although inhibition remained at a satisfactory level (Yu, Wang, Shuian, et al., 2023).

### 3.6 Toxicity of DPP-IV inhibitory peptides in Caco-2 cells

**Fig. 5:**
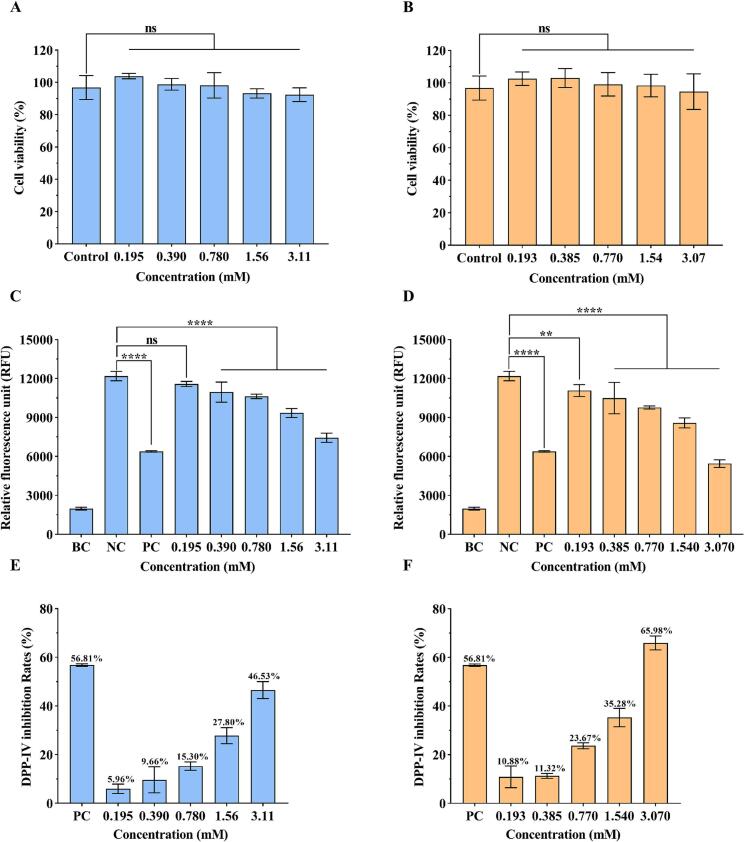
A: Effects of peptides on the growth of Caco-2 cells. (A, B) A: GPFPLL; B: LPYPY. Relative fluorescence unit of Caco-2 cells exposed to various peptide concentrations. (C, D) C: GPFPLL D: LPYPY. (BC denotes the blank control, NC denotes the negative control, and PC denotes the positive control; The mass concentrations of GPFPLL and LPYPY were 2 mg/mL, 1 mg/mL, 0.5 mg/mL, 0.25 mg/mL, and 0.125 mg/mL.) DPP-IV inhibition rate of peptides in Caco-2 cells. (E, F) E: GPFPLL; F: LPYPY.

Caco-2 cells are often used to investigate the inhibitory activity and bioavailability of peptides that inhibit DPP-IV (Lacroix, Chen, Kitts, & Li-Chan, 2017; Pei et al., 2022; Wang, Xie, & Li, 2019). A cytotoxicity assay was performed to assess the potential toxicity of the peptides to the cells. The viability of Caco-2 cells after exposure to GL-6 and LY-5 for 24 h was determined using the CCK-8 method. Neither GL-6 nor LY-5 exhibited significant cytotoxicity to Caco-2 cells at various concentrations (GL-6: 0.195, 0.390, 0.780, 1.56, and 3.11 mM; LY-5: 0.193, 0.385, 0.770, 1.54, and 3.07 mM) (Fig. 5A, B). Therefore, these concentrations were deemed safe for subsequent DPP-IV activity assays as they did not interfere with cell viability.

### 3.7 DPP-IV inhibitory peptide activity in Caco-2 cells

The inhibitory activity of DPP-IV by GL-6 and LY-5 in Caco-2 cells was examined using the fluorescent substrate Gly-Pro-AMC. Because Gly-Pro-AMC is selectively metabolized by DPP-IV to generate AMC fluorophores, the relative fluorescence unit (RFU) decreases as the peptide concentration increases (Fig. 5C, D) (Mu et al., 2024). When the concentration of sitagliptin was 1.0 μM, the inhibition was 56.81 % ± 0.51 %, which was comparable to the results obtained by Lammi et al. (Lammi et al., 2018). As depicted in Fig. 5E, F, the inhibitory effects of GL-6 and LY-5 on DPP-IV exhibited a dose-dependent increase. When the concentration of each peptide was 2 mg/mL, the DPP-IV inhibition rates by GL-6 and LY-5 were 46.53 % ± 3.48 % and 65.98 % ± 2.87 %, respectively. These results suggested that both GL-6 and LY-5 exhibited DPP-IV inhibitory activity in Caco-2 cells. However, it was observed that the inhibitory effects of GL-6 and LY-5 in Caco-2 cells were lower than those in the *in vitro* experiments. This could be ascribed to the presence of brush border enzymes outside the cell that degrade the peptides (Caron, Domenger, Dhulster, Ravallec, & Cudennec, 2017). In addition, the barrier function of the cell membrane and the transport efficiency of the peptides both impact the peptide bioavailability (Yu et al., 2023). Three DPP-IV inhibitory peptides screened from walnut protein by Mu et al. and four screened from chickpea protein by Zan et al. exhibited similar behavior (Mu et al., 2024) (Zan et al., 2023). Through the daily diet of goat milk, peptide concentration perhaps cannot reach the concentration of IC_50_. It still has the potential to reduce blood glucose by inhibiting the activity of DPP-IV. These findings underscore the significant potential of goat milk as a functional food for the prevention of T2DM, enrich the bioactive peptide library associated with goat milk, and enhance the resource utilization of this dairy product. Notably, in comparison with synthetic DPP-IV inhibitors, food-derived peptides possess advantages in safety, biocompatibility, and suitability for prolonged consumption. In future studies, we will plan to conduct further investigations *via in vivo* experiments.

## 4 Conclusion

In conclusion, two candidate competitive DPP-IV inhibitory peptides, GPFPLL and LPYPY, derived from goat milk powder digestive solutions, were successfully identified using molecular docking and MD simulations. Significant DPP-IV inhibition by both GPFPLL and LPYPY was confirmed through *in vitro* cell and cell-free experiments. Our study not only proposes an efficient and effective strategy for virtual screening of DPP-IV inhibitory peptides but also offers valuable insights for the development of novel hypoglycemic peptides.

## CRediT authorship contribution statement

**Kuo Dang:** Writing – original draft, Software, Methodology, Investigation, Formal analysis. **Jing Lan:** Software, Methodology, Investigation. **Yanli Wang:** Visualization, Validation, Supervision, Investigation. **Daodong Pan:** Validation, Supervision, Investigation. **Lihui Du:** Software, Methodology, Investigation. **Shikun Suo:** Supervision, Software, Methodology. **Yali Dang:** Writing – review & editing, Visualization, Validation, Supervision, Project administration, Funding acquisition, Data curation. **Xinchang Gao:** Writing – review & editing, Supervision, Software, Project administration, Investigation.

## Declaration of competing interest

The authors declare that they have no known competing financial interests or personal relationships that could have appeared to influence the work reported in this paper.

## Data Availability

Data will be made available on request.

## Appendix A Supplementary data

Supplementary material Image 1
