# Supplementary material for: Screening and evaluation of novel DPP-IV inhibitory peptides in goat milk based on molecular docking and molecular dynamics simulation
Source: Food Chem X. 2025 Jan 30;25:102217. doi: 10.1016/j.fochx.2025.102217 (PMC11838108; doi:10.1016/j.fochx.2025.102217)
Supplement: Supplementary file 1 — Supplementary material [file mmc1.docx]

**Screening and Evaluation of Novel DPP-IV Inhibitory Peptides in Goat Milk Based on Molecular Docking and Molecular Dynamics Simulation**

Kuo Dang^a^, Jing Lan^a^, Yanli Wang^a^, Daodong Pan^a^, Lihui Du^a^, Shikun Suo^a^, Yali Dang^a,^**^*^**, Xinchang Gao^b,*^

**Author Affiliation(s)**

^a^ College of Food Science and Engineering, Ningbo University, Ningbo, 315211, Zhejiang, China

^b^ Institute of Drug Discovery Technology, Ningbo University, Ningbo, 315211, China

*Corresponding author:

Yali Dang, College of Food Science and Engineering, Ningbo University, Ningbo 315211, Zhejiang, China. E-mail addresses: dangyali@nbu.edu.cn.

Xinchang Gao, Institute of Drug Discovery Technology, Ningbo University, Ningbo, 315211, China. E-mail addresses: [gaoxinchang@nbu.edu.cn](mailto:gaoxinchang@nbu.edu.cn).

Captions:

Figure 1. The inhibition rate of DPP-IV in the digestive solution of goat milk at various concentrations (< 3 kDa permeate).

Figure 2. Synthetic materials of peptides (HPLC) (A-D). A: GPFPLL B: LPYPY C: GPFPILN D: RPWR.

Figure 3. Synthetic materials of peptides (MS) (A-D). A: GPFPLL B: LPYPY C: GPFPILN D: RPWR.


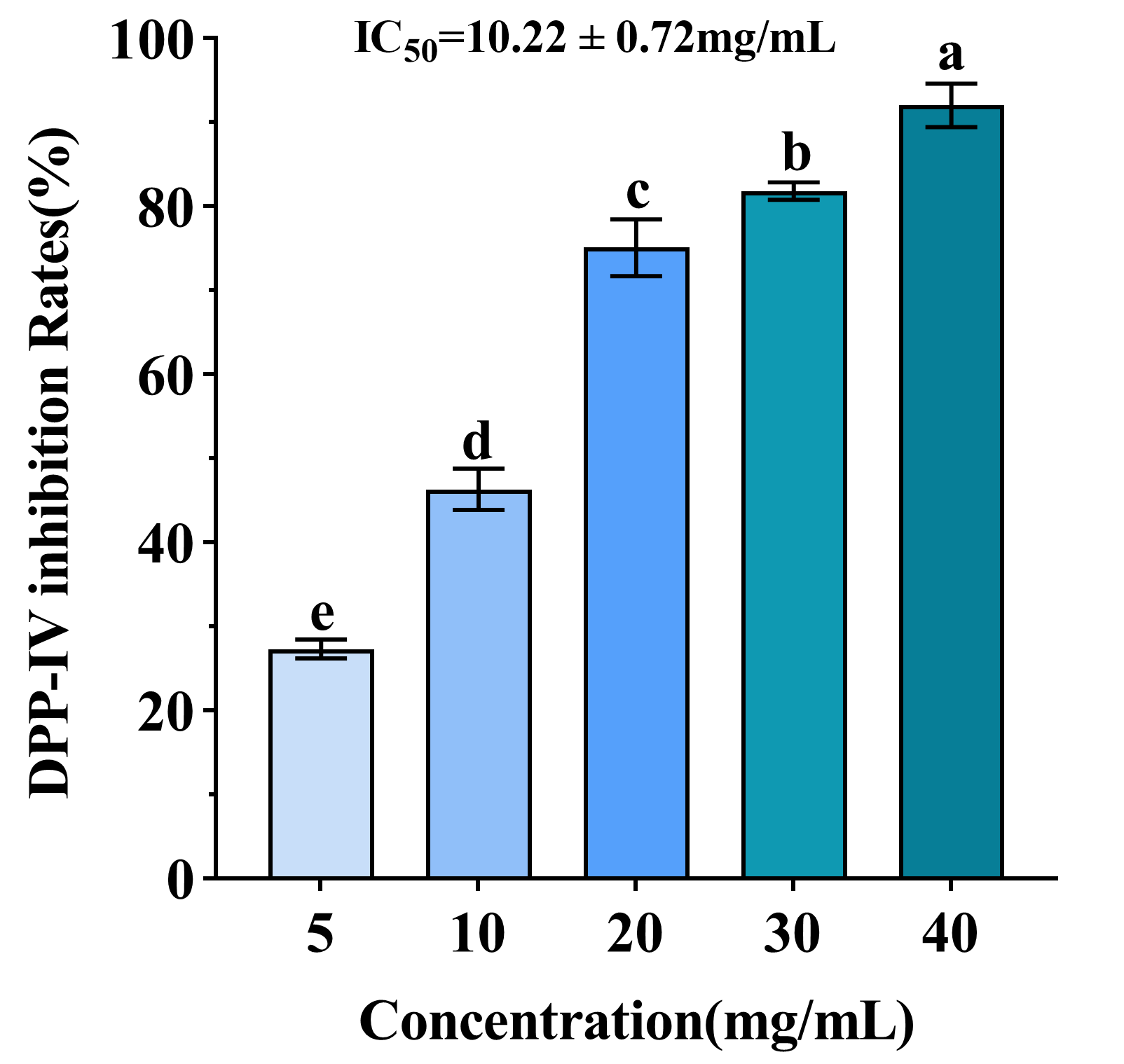


Figure 1. The inhibition rate of DPP-IV in the digestive solution of goat milk at various concentrations (< 3kDa permeate).





Figure 2. Synthetic materials of peptides (HPLC) (A-D). A: GPFPLL B: LPYPY C: GPFPILN D: RPWR.





Figure 3. Synthetic materials of peptides (MS) (A-D). A: GPFPLL B: LPYPY C: GPFPILN D: RPWR.
